# Genome analysis of *Phytophthora cactorum* strains associated with crown- and leather-rot in strawberry

**DOI:** 10.3389/fmicb.2023.1214924

**Published:** 2023-07-03

**Authors:** Anupam Gogoi, Simeon L. Rossmann, Erik Lysøe, Arne Stensvand, May Bente Brurberg

**Affiliations:** ^1^Department of Plant Sciences, Faculty of Biosciences (BIOVIT), Norwegian University of Life Sciences (NMBU), Ås, Norway; ^2^Division of Biotechnology and Plant Health, Norwegian Institute of Bioeconomy Research (NIBIO), Ås, Norway

**Keywords:** oomycete, PacBio sequel II sequencing, elicitors, RxLRs, Crinklers, effectors

## Abstract

*Phytophthora cactorum* has two distinct pathotypes that cause crown rot and leather rot in strawberry (*Fragaria* × *ananassa*). Strains of the crown rot pathotype can infect both the rhizome (crown) and fruit tissues, while strains of the leather rot pathotype can only infect the fruits of strawberry. The genome of a highly virulent crown rot strain, a low virulent crown rot strain, and three leather rot strains were sequenced using PacBio high fidelity (HiFi) long read sequencing. The reads were *de novo* assembled to 66.4–67.6 megabases genomes in 178–204 contigs, with N50 values ranging from 892 to 1,036 kilobases. The total number of predicted complete genes in the five *P. cactorum* genomes ranged from 17,286 to 17,398. Orthology analysis identified a core secretome of 8,238 genes. Comparative genomic analysis revealed differences in the composition of potential virulence effectors, such as putative RxLR and Crinklers, between the crown rot and the leather rot pathotypes. Insertions, deletions, and amino acid substitutions were detected in genes encoding putative elicitors such as beta elicitin and cellulose-binding domain proteins from the leather rot strains compared to the highly virulent crown rot strain, suggesting a potential mechanism for the crown rot strain to escape host recognition during compatible interaction with strawberry. The results presented here highlight several effectors that may facilitate the tissue-specific colonization of *P. cactorum* in strawberry.

## 1. Introduction

*Phytophthora cactorum* is an economically important soil-borne oomycete pathogen that infects more than 200 plant species, a majority of which are herbaceous and woody plants (Erwin and Ribeiro, 1996; Chen et al., 2023). The pathogen has two distinct pathotypes that causes crown rot and/or leather rot in strawberry (*Fragaria* × *ananassa* Duch)(van der Scheer, 1971; Harris and Stickels, 1981; Eikemo et al., 2004). Both diseases cause substantial yield losses in strawberry production; in addition the leather rot affects post-harvest processing of strawberry fruits (Harris and Stickels, 1981; Ellis and Grove, 1998; Stensvand et al., 1999; Pánek et al., 2022). The crown rot pathotype can cause disease in both crown and fruit tissues, while the leather rot pathotype causes disease only in the strawberry fruit. Strains of *P. cactorum* from other hosts such as almond, apple, peach, rhododendron and silver birch can cause leather rot in strawberry, but not crown rot (Eikemo et al., 2004). The use of fungicides such as methyl bromide, mefenoxam, and metalaxyl are effective against *P. cactorum* (Ellis et al., 1998; De Cal et al., 2004; Maas, 2014); however, the phasing-out of methyl bromide due to its impact on stratospheric ozone, development of fungicide resistance, and strict regulation on the use of metalaxyl in agriculture due its potential high risk on human and animal health have provided challenges to control both the diseases in strawberry (Hrelia et al., 1996; European Food Safety Authority, 2015; Marin et al., 2021; Ali et al., 2022).

The pathogenicity of *P. cactorum* on strawberry crowns varies among strains and depends on host cultivars (Pettitt and Pegg, 1994; Eikemo et al., 2003; Nellist et al., 2021). For instance, several strawberry cultivars and their wild relatives *F. vesca*, *F. chiloensis*, and *F. virginiana* show varying degrees of susceptibility to both crown rot and leather rot (Parikka, 2003; Shaw et al., 2006; Eikemo and Stensvand, 2015). In a previous study, no correlation was observed between resistance to crown rot and resistance to leather rot among a selected group of strawberry genotypes (Eikemo and Stensvand, 2015).

*Phytophthora cactorum* reproduces with asexual sporangia that release zoospores upon physical contact with water and with oospores resulting from sexual reproduction. The pathogen is homothallic which limits the possibility of crossing between strains and among pathotypes (Blackwell, 1943; Goodwin, 1997). The genetic distinction between crown rot and leather rot pathotypes have mainly been studied through the use of polymorphic markers (Hantula et al., 2000; Eikemo et al., 2004). Based on random amplified microsatellite markers, no genetic differentiation was observed between strains from leather rot and crown rot of strawberry. However, using amplified fragment length polymorphism markers, Eikemo et al. (2004) showed that *P. cactorum* strains designated as crown rot and leather rot were genetically different, and that crown rot strains had low genetic diversity. This was later confirmed in a study investigating evolutionary relationships within the *Phytophthora cactorum* species complex in Europe (Pánek et al., 2016). The genetic difference between crown rot and leather rot pathotypes of *P. cactorum* could be explained by different sexual behavior in various mating population (Pánek et al., 2021).

The feasibility and cost-effectiveness of sequencing whole genomes have provided a framework to understand disease effector repertoires in several *Phytophthora* species (Armitage et al., 2018; Zhang et al., 2019; Lee et al., 2020). Species within *Phytophthora* are known to secret an arsenal of effectors, which play diverse roles in plant colonization (McGowan and Fitzpatrick, 2017; Wang et al., 2019). These effectors may localize to the intercellular spaces of the host (apoplastic effectors) or translocate into different cytoplasmic compartments of the host cell (cytoplasmic effectors). Apoplastic effectors include enzyme inhibitors such as serine and cysteine protease inhibitors, carbohydrate-active enzymes (CAZymes) and cutinases that have been proposed to degrade plant cell wall components, elicitins (sterol-binding proteins), cellulose-binding elicitor lectin (CBEL), Nep1-like proteins, phytotoxins, and PcF (*Phytophthora cactorum*-*Fragaria*)-like small cysteine-rich proteins (SCRs) (Orsomando et al., 2001; Kamoun, 2006; Raaymakers and Van den Ackerveken, 2016). Two key groups of cytoplasmic effectors, RxLRs and Crinklers (CRN for crinkling and necrosis) have been identified in *Phytophthora* spp. The canonical motifs, RxLR-dEER of RxLR effectors and LFLAK-HVLV of CRN effectors that are present downstream of a signal peptide in the N-terminus are known to be involved in translocation into the host. The highly variable C-terminal domain of these effectors is dedicated to diversifying host targets and helps in modulating plant defense responses (Win et al., 2007; Haas et al., 2009; Armitage et al., 2018).

Plants employ diverse immune receptors to sense microbe- or pathogen- associated molecular patterns (MAMPs or PAMPs) and virulence effectors of pathogens, to trigger host immunity (Jones and Dangl, 2006). These includes plasma membrane localized receptor-like kinases (RLKs) and receptor-like proteins (RLPs), also known as pattern recognition receptors (PRRs) and intracellular immune receptors such as nucleotide-binding and leucine-rich repeat proteins (NLRs). The recognition of molecular patterns (MAMPs or PAMPs) by host PRRs activates pattern-triggered immunity (PTI), whereas recognition of effectors by host NLRs activates effector-triggered immunity (ETI) in plants (Dodds and Rathjen, 2010; Boutrot and Zipfel, 2017). Several elicitors from *Phytophthora* species such as CBEL, elicitins, transglutaminases, and a secreted protein OPEL from *P. parasitica* act as PAMPs that are sensed by PRRs, and activate PTI (Raaymakers and Van den Ackerveken, 2016; Boutrot and Zipfel, 2017). PTI responses in plants can mediate a broad spectrum and durable resistance against several *Phytophthora* species (Du et al., 2015). Similarly, the recognition of *Phytophthora* effectors such as RxLRs by NLRs activate ETI, resulting in localized cell death to restrict growth of the pathogen (Wu et al., 2017). However, the virulent strains of a pathogen species can evade the recognition capabilities of host proteins during a so-called compatible plant-pathogen interaction. This can be accomplished through sequence variations, gene losses and/or transcriptional polymorphisms of the target ligand (Gilroy et al., 2011; Na and Gijzen, 2016). In addition, changes in copy number of effector genes have been proposed as a contributing factor for pathogen fitness (Qutob et al., 2009; Knaus et al., 2019).

Previously sequenced genomes of *P. cactorum* have been reported with different genome sizes and compositions (Grenville-Briggs et al., 2017; Armitage et al., 2018; Yang et al., 2018a). The Illumina-based genome assemblies of *P. cactorum* strains 10300 and LV007 were based on strains from infected strawberry (Armitage et al., 2018) and European beech (*Fagus sylvatica*; Grenville-Briggs et al., 2017), while the PacBio-based genome assembly of *P. cactorum* was of strain from infected Chinese ginseng (*Panax notoginseng*; Yang et al., 2018a), designated as “Yunnan” in this work. Recently, a PacBio RS-II-based and several Illumina-based genome assemblies of *P. cactorum* from infected strawberry and apple (*Malus* × *domestica*) have been reported (Nellist et al., 2021). The latter study showed that *P. cactorum* from strawberry and apple hosts comprises distinct phylogenetic lineages. They identified several candidate RxLR effectors proposed to be involved in host specialization on strawberry crown and apple tissues. The available transcriptome datasets of *P. cactorum* are also valuable resources for understanding the role of effectors during infection in hosts (Chen et al., 2014, 2018; Nellist et al., 2021).

In the study presented here, we aimed to detect specific genetic differences between the *P. cactorum* crown rot pathotype and the leather rot pathotype, and to understand the mechanisms of tissue specific colonization of the *P. cactorum* pathotypes. Hence, we sequenced the genomes of crown rot and leather rot strains using the latest PacBio Sequel II system, which provides single molecule high-fidelity (HiFi) long reads from the circular consensus sequencing (CCS) mode and a base-resolution with >99.8% single molecule read accuracy (Wenger et al., 2019). The use of long HiFi reads can facilitate *de novo* assembly of repetitive genomes and accurate representation of structural variants, small insertions/deletions and single nucleotide polymorphisms (Hon et al., 2020). We report five new genome assemblies of *P. cactorum* with high contiguity and accuracy. In addition, we report several new candidate genes that have a potential role in tissue-specific colonization of *P. cactorum* in strawberry.

## 2. Materials and methods

### 2.1. Isolation of *Phytophthora cactorum*

Plants with visible crown rot or leather rot symptoms were collected from different strawberry and apple plantations in Norway. Infected rhizomes (crowns) or fruits were rinsed in tap water for 20 min to remove superficial soil and debris. The samples were surface sterilized with 70% ethanol for 10 s, followed by treatment with 0.5% sodium hypochlorite for 90 s, and finally rinsed three times with distilled water. Infected plant parts were sliced into rectangular cubes using a sterilized scalpel and were placed on solid PARP medium (Jeffers and Martin, 1986). The plates were incubated in the dark at room temperature (20°C) for 5–7 days and were examined for mycelium growth and typical sporulation patterns (sporangia and oospores) of *P. cactorum* under light microscopy.

### 2.2. Plant material and pathogen inoculation

Eleven *P. cactorum* strains were tested for their ability to cause (1) crown rot in the rhizome of the susceptible strawberry cultivars Polka and Korona; and (2) leather rot on unripe fruits of Korona. Five of the *P. cactorum* strains from naturally infected strawberry fruits (252360, 252362, 252363, 252364, 252365), and one strain from apple fruit (251539) were tested for their ability to cause crown rot in the highly susceptible cultivar Polka, to identify the leather rot pathotype. The four strains from naturally infected strawberry rhizomes (251171, 251615, 251616, 251683) were tested for their ability to cause crown rot and leather rot in “Korona” (moderately resistant to *P. cactorum*; Eikemo and Stensvand, 2015), together with previously isolated crown rot strains 10300 (Armitage et al., 2018) and P414 (Nellist et al., 2021). A moderately resistant cultivar was used in this case to study the aggressiveness of different *P. cactorum* strains based on the degree of necrosis in the rhizome.

Strawberry plants were grown in a greenhouse at 18°C with a 16/8 h light/dark regime. For pathogen inoculation, each *P. cactorum* strain was grown on 10% V8 (vegetable juice) agar plates (Jeffers, 2015) and incubated at room temperature (20°C) for 2 weeks to promote sporangia formation. Zoospore suspensions were prepared using autoclaved pond water as previously described by Eikemo et al. (2000). Plant tissue was gently wounded using a sterilized scalpel (for rhizome inoculation) or a pipette tip (for fruit inoculation) and inoculated with 2 × 10^5^ zoospores of *P. cactorum*. Subsequently, the rhizome infection was followed for 4 weeks, and disease symptoms were scored 8-1, where scores 8, 7, 6, 5 represent plants that died due to wilting during the first, second, third, and fourth week after inoculation with *P. cactorum*, respectively. Plants that survived 4 weeks after inoculation were bisected longitudinally using a sterile scalpel and scored based on the degree of necrosis in the rhizome (4 = clear necrosis covering at least 50% of the rhizome area, 3 = small patches of necrosis, 2 = minor dark brown speckles, 1 = no symptoms) as described by Bell et al. (1997). Inoculated fruits were given a binary score (0 = no infection and 1 = infection).

### 2.3. Isolation of genomic DNA from *Phytophthora cactorum*

Genomic DNA was isolated from the mycelium of five *P. cactorum* strains 251539, 251616, 251683, 252360, and 252365 using a modified phenol-chloroform extraction protocol for *Phytophthora* (Joint Genome Institute, 2021). Briefly, 5 g of mycelial mat grown in 100 mL V8 broth was collected using a Miracloth (EMD Millipore, Merck, Germany) and ground using a pre-cooled mortar and pestle, plus liquid N_2._ Ten mL of extraction buffer (0.2 M of tris(hydroxymethyl)aminomethane, pH 8.5, 0.25 M sodium chloride, 25 mM ethylenediaminetetraacetic acid, and 0.5% sodium dodecyl sulfate) was added to the mycelial powder and mixed well using a pipette. Subsequently, 20 μL of 100 μg/mL RNase A (Qiagen, Germany) was added and the lysate was incubated at 65°C for 10 min. Following the incubation, 7 mL of tris-equilibrated phenol, pH 8.0 and 3 mL of chloroform was added, gently mixed, and incubated at room temperature (20°C) for 1 h. Samples were centrifuged at 6,000 g for 15 min at 4°C. The aqueous phase was added to an equal volume of chloroform, gently mixed, and centrifuged at 6,000 g for 5 min at 4°C. After centrifugation, the aqueous phase was transferred to a pre-cooled 50 mL centrifuge tube (VWR, United States), gently mixed with 0.6 volume of chilled isopropanol, incubated on ice for 30 min, and centrifuged at 6,000 g for 30 min at 4°C to recover the precipitated DNA. The DNA pellet was washed three times with 70% ethanol, air dried and dissolved in 300 μL of nuclease free water. The DNA concentration was measured using the NanoDrop^™^ 2000 Spectrophotometer (ThermoFisher Scientific, United States). An aliquot of the isolated DNA was analyzed using agarose gel electrophoresis run at 30 V for 16 h to assess the quality of the intact genomic DNA for sequencing.

### 2.4. Genome sequencing

Twenty micrograms (50 ng/μL) of the isolated DNA for each sample was sent to the Norwegian Sequencing Centre, Oslo, for library preparation and sequencing. All five *P. cactorum* genomic libraries were prepared according to the manufacturer’s protocol for Multiplexing SMRTbell libraries using the SMRTbell Express Template Prep Kit 2.0 (Pacific Biosciences, United States). DNA was fragmented to 20 kb fragments using Megaruptor^®^ 3 (Diagenode s.a., Belgium) and size selected using Bluepippin with 15 kb cut-off. The libraries were sequenced on one 8 M SMRT cell using the Sequel II Binding Kit 2.0 with the sequencing chemistry v2.0 from the PacBio Sequel II (Pacific Biosciences, United States). Reads were demultiplexed using Demultiplex Barcodes pipeline on SMRT Link v 8.0.0.80529 (SMRT Tools v 8.0.0.80502).

### 2.5. *De novo* genome assembly, gene prediction, and genome completeness

Raw subreads were trimmed for adapters and low-quality regions and processed to obtain error corrected CCS (circular consensus sequencing) reads. The high-quality CCS (HiFi) reads (Phred quality score ≥ Q20) with a minimum predicted accuracy 0.9 were used for *de novo* genome assembly using the automated Flye v2.8.2 pipeline (Kolmogorov et al., 2019) in OmicsBox (2019). Because of the high-quality of the sequenced reads and to increase the possibility of detecting true sequence diversity among the five strains of *P. cactorum*, *de novo* genome assembly was performed. Assembly statistics were generated using QUAST v5.0.2 (Gurevich et al., 2013) and BUSCO statistics were obtained using BUSCO v4.1.2 (Simão et al., 2015) with the eukaryota_odb10 database (updated on 2020-03-06). The repeat contents of the assembled genomes were obtained using RepeatMasker Open-4.0 (Smit et al., 2015) and RepeatMasker combined library of Dfam_Consensus and RepBase v20170127 (Bao et al., 2015; Hubley et al., 2016). Genes were predicted using the assembled contigs of each *P. cactorum* genomes in the *ab initio* mode of Augustus v3.3.3 (Stanke et al., 2008). Augustus was trained using predicted protein sequences from *P. cactorum* strain 10300 (Armitage et al., 2018).

### 2.6. Functional annotations and secretome prediction

The predicted proteome of the *P. cactorum* strains were annotated using a combination of BLASTP (Altschul et al., 1990) against the NCBI non-redundant (Nr) database and InterProScan (Zdobnov and Apweiler, 2001) to assign individual sequences with gene ontology (GO) terms (Ashburner et al., 2000) using Blast2GO (Götz et al., 2008).

Secreted proteins of *P. cactorum* (secretome) were predicted using a combination of tools that included SignalP v5.0 (Armenteros et al., 2019b) to detect signal peptide cleavage sites, Phobius (Käll et al., 2007) to predict transmembrane domains and signal peptide cleavage sites, TargetP v2.0 (Armenteros et al., 2019a) to predict the presence of mitochondrial transit peptides and signal peptide cleavage sites. The presence of endoplasmic reticulum retention signals (KDEL or HDEL motifs) were predicted as previously described by Evangelisti et al. (2017) using PROSITE-Scan (de Castro et al., 2006). No subcellular localization of “extracellular” as criterion for predicting secretion was considered, as recommended for oomycetes (Sperschneider et al., 2015). A protein was defined as “secreted” if it contained a signal peptide cleavage site within the first 70 amino acids in the N-terminus, had no more than one transmembrane domain, as recommended for oomycetes (Sperschneider et al., 2015), and had no mitochondrial localization or endoplasmic reticulum retention signals. The predicted secretome was used to predict the apoplastic proteins using ApoplastP (Sperschneider et al., 2018).

Genes encoding RxLR and CRN effectors were predicted using the six-frame translation of open reading frames (ORFs) with a minimum cutoff of 100 aa using *getorf* from EMBOSS (Rice et al., 2000). The translated ORFs were used to search for canonical RxLR-EER motifs (for RxLR effectors) and the LFLAK-HVLV motifs (for CRN effectors) in R using the *effectR* package (Tabima and Grünwald, 2019). Predicted RxLR and CRN effector proteins were searched for signal peptides in the N-termini using SignalP v5 and annotated using BLASTP (Altschul et al., 1990) against the NCBI non-redundant (Nr) database. Candidate effectors with a predicted signal peptide in the N-terminus and no more than one transmembrane domain were considered high-confidence effectors.

### 2.7. Gene orthology analysis

Orthologues of the predicted proteome of the *P. cactorum* strains sequenced in this study (251539, 251616, 251683, 252360, and 252365), the *P. cactorum* 10300 strain (Armitage et al., 2018), the Yunnan strain (Yang et al., 2018a), and predicted proteomes of publicly available *P. capsici* LT1534 v11.0 (Lamour et al., 2012), *P. infestans* T30-4 (Haas et al., 2009), *P. palmivora* ZC01 v1 (Gil et al., 2020), *P. parasitica* INRA-310 (NCBI dataset, GCA_000247585.2 PP_INRA-310_V2, unpublished), and *P. sojae* P6497 v3.0 (Tyler et al., 2006) were identified using OrthoFinder v2.4.0 (Emms and Kelly, 2019). The concatenated orthologues (one ortholog per orthogroup per genome) shared by the *P. cactorum* strains and other *Phytophthora* spp. were used to construct a phylogenomic tree using the maximum-likelihood method implemented in the phangorn package (Schliep, 2011), with the Le Gascuel (LG) model for amino acid substitutions (Le and Gascuel, 2008). Predicted secreted proteins of the sequenced *P. cactorum* strains were compared using Orthovenn2 with expectation (E) value equal to 1e-10 and inflation value equal to 1.5 (default) to obtain higher resolution between groups (Xu et al., 2019). Predicted RxLR and CRN proteins were clustered using the OrthoFinder v2.4.0 and visualized in R using the Tidyverse package collection (Wickham et al., 2019).

### 2.8. Read mapping and variant calling

The HiFi reads were aligned to the *de novo* assembled genome of *P. cactorum* crown rot strain 251616 (used as reference) using pbmm2 v1.3.0^1^ wrapper for minimap2 aligner (Li, 2018). The output bam files were indexed using samtools v1.10 (Danecek et al., 2021), and variants were called in the HiFi reads using DeepVariant v1.0.0 with a pre-defined model (--model_type = PACBIO; Poplin et al., 2018). Phasing of DNA variants was performed using WhatsHap v1.0 (Patterson et al., 2015). The phased variants (SNPs) were filtered and sorted using VCFtools v0.1.15 (Danecek et al., 2011). The resulting phased SNPs and indels (unphased) were annotated, and variant effects were predicted using SnpEff (Cingolani et al., 2012). To perform variant annotations, a manual database was built using the general feature format (GFF3) file of the predicted genes of strain 251616. High-quality SNPs and indels with Phred quality score ≥ 20 were used for downstream analysis. Pairwise alignment of selected protein-coding genes from strain 251616 and a representative leather rot strain 251539 was done using LASTZ v1.04.03^2^ (Harris, 2007). The rate of nonsynonymous and synonymous substitutions (dN/dS or ω) was calculated using KaKs_Calculator 2.0 (Wang et al., 2010). Genes with dN/dS ratio greater than one and *p* < 0.05 (calculated by the Fisher’s extract test) were considered under positive selection.

## 3. Results

### 3.1. Pathogenicity of *Phytophthora cactorum* strains in strawberry

**Figure 1:**
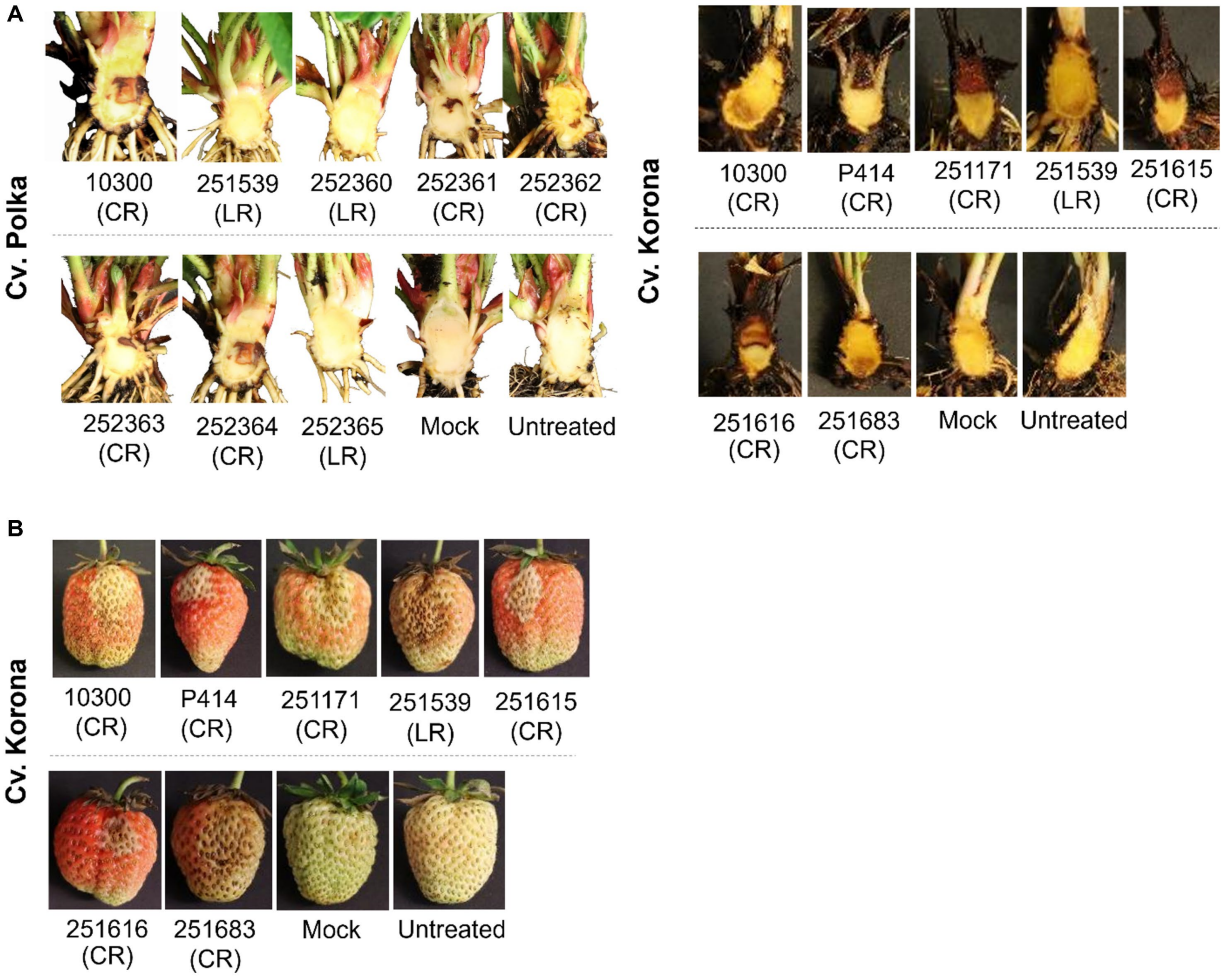
*Phytophthora cactorum* strains show tissue-specific colonization in strawberry. **(A)** Crown rot symptoms after inoculation with different strains of *P. cactorum* into the rhizome of a highly susceptible cultivar “Polka” (left panel) and into the rhizome of a moderately resistant cultivar “Korona” (right panel). Photos were taken 4 weeks after inoculation with *P. cactorum* zoospores. **(B)** Leather rot symptoms after inoculation with different strains of *P. cactorum* into strawberry fruits. A typical fruit discoloration on the infected fruits was observed within 4–6  days after inoculation (dai) with *P. cactorum*. Each photo is a representative of 5 biological replicates at 4 dai. Negative controls tissues were wounded and inoculated with autoclaved pond water (mock). “CR” indicate crown rot pathotype, whereas “LR” indicate leather rot pathotype.

Seven *P. cactorum* strains that were isolated from strawberry fruits and apple fruit were tested for virulence in the rhizome (crown) by inoculation in the highly susceptible cultivar Polka. Four of these (252361, 252362, 252363, 252364) developed brown necrotic lesions in the rhizome, and were therefore classified as crown rot (CR) pathotype. In contrast, the strain from apple fruit 251539, and two other strawberry fruit strains 252360 and 252365, did not develop crown rot symptoms (Table 1; Figure 1A). Consequently, the latter three *P. cactorum* strains were classified as leather rot (LR) pathotype.

**Table 1:** Overview of the *Phytophthora cactorum* strains used in this study.

Strain	Plant source (cultivar)	Plant part isolated from^a^	Country of origin	Disease score (Mean ± SE)			Pathotype^d^	Previously reported
				Crown rot^b^		Leather rot^c^		
				“Korona”	“Polka”	“Korona”		
251539	Apple	Fruit	Norway	1.0 ± 0.0	1 ± 0.0	1 ± 0.0	LR	
252360	Strawberry (Rumba)	Fruit	Norway	–	1 ± 0.0	–	LR	
252361	Strawberry (Rumba)	Fruit	Norway	–	2 ± 0.4	–	CR	
252362	Strawberry (Rumba)	Fruit	Norway	–	1.5 ± 0.3	–	CR	
252363	Strawberry (Korona)	Fruit	Norway	–	1.9 ± 0.4	–	CR	
252364	Strawberry (Korona)	Fruit	Norway	–	1.6 ± 0.3	–	CR	
252365	Strawberry (Korona)	Fruit	Norway	–	1.1 ± 0.1	–	LR	
10300	Strawberry	Rhizome	Norway	1.8 ± 0.6	2 ± 0.3	1 ± 0.0	CR	Armitage et al. (2018)
P414	Strawberry	Rhizome (crown)	United Kingdom	3 ± 1.1	–	1 ± 0.0	CR	Nellist et al. (2021)
251171	Strawberry (Sonata)	Rhizome	Norway	2.4 ± 1.1	–	1 ± 0.0	CR	
251615	Strawberry (Sonata)	Rhizome	Norway	3.4 ± 1.5	–	1 ± 0.0	CR	
251616	Strawberry (Korona)	Rhizome	Norway	5.4 ± 1.0	–	1 ± 0.0	CR	
251683	Strawberry (Korona)	Rhizome	Norway	1.2 ± 0.2	–	1 ± 0.0	CR	

a The strain P414 was previously isolated from strawberry crown which is technically rhizome tissue.

b Five individual plants of each strawberry cultivar (Korona and/or Polka) were inoculated with *P. cactorum* zoospores (2 mL of 2 × 10^5^ zoospores). Disease scoring was performed as previously described by Bell et al. (1997). Data represent mean disease score of two independent inoculation experiments. SE, standard error of mean. ‘-’ indicates not tested.

c Five fruits were inoculated with *P. cactorum* zoospores and were given a binary score (1 = infection and 0 = no infection). ‘-’ indicates not tested.

d CR and LR represent crown rot and leather rot pathotypes.

The four *P. cactorum* strains isolated from rhizomes with symptoms of crown rot, 251171, 251615, 251616, and 251683, were tested for virulence by inoculation into the rhizome of the moderately resistant cultivar Korona, together with two control crown rot strains (10300 and P414). As expected, all strains developed necrosis in the rhizome, but symptoms varied in severity (Table 1; Figure 1A). Strain 251616 developed a severe necrosis in the strawberry rhizome and is hereafter referred to as high virulence crown rot strain, while the strain 251683 developed few necrotic lesions and is therefore referred to as low virulence crown rot strain (Table 1; Figure 1A). All these strains also developed leather rot on strawberry fruit 4 days after inoculation of berries (Figure 1B).

### 3.2. Highly contiguous genome assemblies of *Phytophthora cactorum* strains

The genomes of two CR strains and three LR strains were sequenced. A total of approximately 3.9 million raw reads were obtained with an average read length of 73 kilobases (kb). High-quality CCS (HiFi) reads used for *de novo* genome assemblies of the five *P. cactorum* strains resulted in genome sizes of 66.4 to 67.6 Mb, each in 178 to 203 contigs with N50 values ranging from 892.1 to 1036.2 kb. The repeat content of the *de novo* assembled *P. cactorum* genomes ranged from 14.5 to 16.1 Mb (mean 15.7 Mb), which constituted a mean of 23.3% of the total genome size. The CR strains of *P. cactorum*, 251616 and 251683, contained slightly more repetitive elements than the LR strains, 251539, 252360, and 252365 (Table 2).

**Table 2:** Overview of the genome assemblies and gene prediction statistics for *Phytophthora cactorum* strains used in this study.

Species	*Phytophthora cactorum*						
Pathotype	Leather rot			Crown rot			
Strain	251539	252360	252365	251683	251616	10300	P414
NGS platform	PacBio SMRT^™^ Sequel II	PacBio SMRT^™^ Sequel II	PacBio SMRT^™^ Sequel II	PacBio SMRT^™^ Sequel II	PacBio SMRT^™^ Sequel II	Illumina	PacBio SMRT^™^ RS-II
Assembler	Flye	Flye	Flye	Flye	Flye	ABySS	SMARTdenovo
Assembly size (Mb)	66.4	67.5	67.5	67.5	67.6	59.3	66.0
Number of contigs	204	178	189	192	202	4,623	194
Largest contigs (kb)	2,445	2,683	2,750	4,546	2,516	301	1,750
N50 (kb)	892	1,036	1,028	968	1,021	56	645
GC content	51%	51%	51%	51%	51%	50%	51%
Repeatmasked (Mb)	14.5	15.9	15.9	16	16	11	19.3
CEGs in assembly^a^	239 (94%)	239 (94%)	239 (94%)	239 (94%)	239 (94%)	239 (94%)	224 (95.7)
Predicted genes (partial genes)^b^	17,398 (3)	17,286 (1)	17,326 (5)	17,318 (1)	17,324 (5)	18,189 (443)	29,552 (−)
CEGs in gene model^c^	244 (96%)	244 (96%)	244 (96%)	244 (96%)	244 (96%)	242 (95%)	–

The previously published genome sequences of *P. cactorum* strain 10300 and P414 from strawberry rhizome is included for comparison (Armitage et al., 2018; Nellist et al., 2021).

a Percentage of the core eukaryotic genes (CEGs) present in genome assembly.

b Gene prediction was performed on the listed assemblies, except P414, using a gene model derived from strain 10300 (Armitage et al., 2018).

c Percentage of the core eukaryotic genes (CEGs) present in the predicted protein-coding genes.

The total number of predicted complete genes in the assembled genomes of the five *P. cactorum* strains ranged from 17286 to 17398 (Supplementary Table S1). All five *P. cactorum* assemblies contained 239 of 255 core eukaryotic genes (CEGs), representing a genome completeness of 94%, which was comparable to the percentage of CEGs (96%) in the predicted proteomes. The near complete set of CEGs suggested overall accurate gene prediction and support the validity of the used model. The percentage of CEGs was similar in the assembly (94–96%) to those of the previously published CR strains 10300 and P414 assembly (Table 2).

### 3.3. Comparative genomic analysis of *Phytophthora cactorum* strains

#### 3.3.1. Phylogenomic analysis

**Figure 2:**
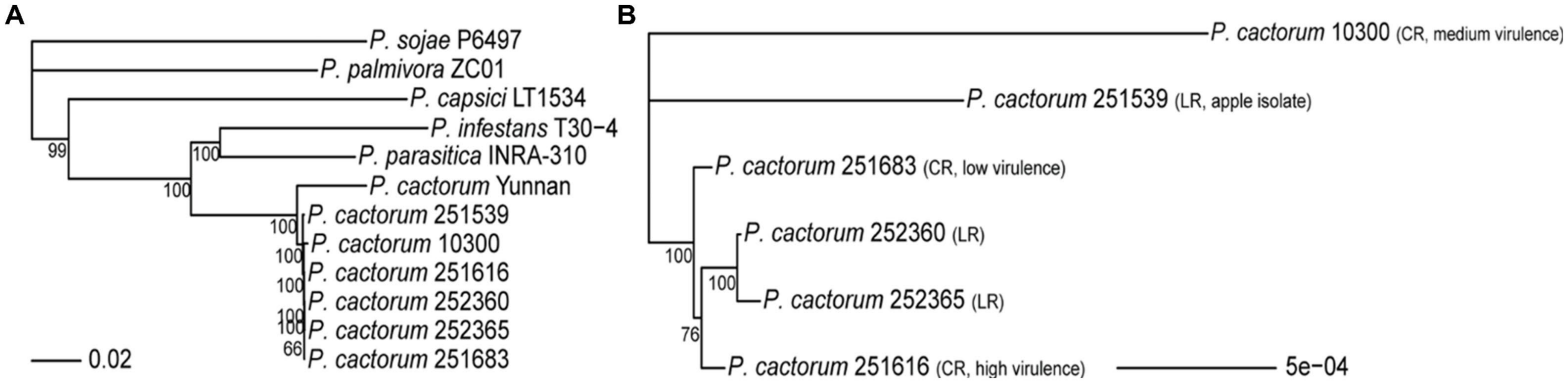
Phylogenomic tree of *Phytophthora cactorum* based on 2031 single-copy orthologous protein sequences. **(A)** Maximum-likelihood phylogenomic tree based on 2031 concatenated single-copy orthologues per genome of the sequenced *P. cactorum* strains (251539, 252360, 252365, 251683, and 251616), the previously published crown rot strain 10300, the “Yunnan” strain as well as other publicly available *Phytophthora* species. **(B)** Maximum-likelihood phylogenomic tree containing only the sequenced crown rot (CR) and leather rot (LR) strains of *P. cactorum*. Nodes were labeled with the percentage of bootstraps (*n* = 10,000) that supported a given node, values lower than 50% are not shown.

The predicted proteomes of the *P. cactorum* strains sequenced in this study were compared to the proteomes of *P. capsici*, *P. infestans*, *P. palmivora*, *P. parasitica*, and *P. sojae*, to identify orthologous protein sequences. A total of 253,402 protein sequences from 12 genomes were assigned to 21,031 orthogroups, which accounted for 96.2% of all predicted proteomes from all investigated *Phytophthora* species. For phylogenomic analysis, 2031 single-copy orthologs that were present in all investigated genomes (one copy per orthogroup) were concatenated into one sequence per genome and used to construct a phylogenomic tree using a maximum-likelihood model and 10,000 bootstraps (Figure 2). The *P. cactorum* strains were highly similar to each other when compared to those of other *Phytophthora* species (Figure 2A). For finer resolution of the differences between CR and LR strains, a phylogenomic tree containing only the *P. cactorum* strains sequenced in this study and the previously published crown rot strain 10300 was constructed (Figure 2B). The two LR strains from strawberry (252360 and 252365) clustered, while the LR strain from apple (251539) was phylogenetically distinct from the strawberry strains. The CR strains 251616 (high virulence) and 251683 (low virulence) were closely related but did not form a stable cluster that was distinct from the strawberry LR strains. Instead, 251616 was closer to the strawberry LR strains than to 251683 in 76% of the bootstraps. The previously sequenced CR strain 10300 (medium virulence) was in a separate clade (Figure 2B).

#### 3.3.2. Orthologous clustering of secreted proteins from *Phytophthora cactorum* strains

**Figure 3:**
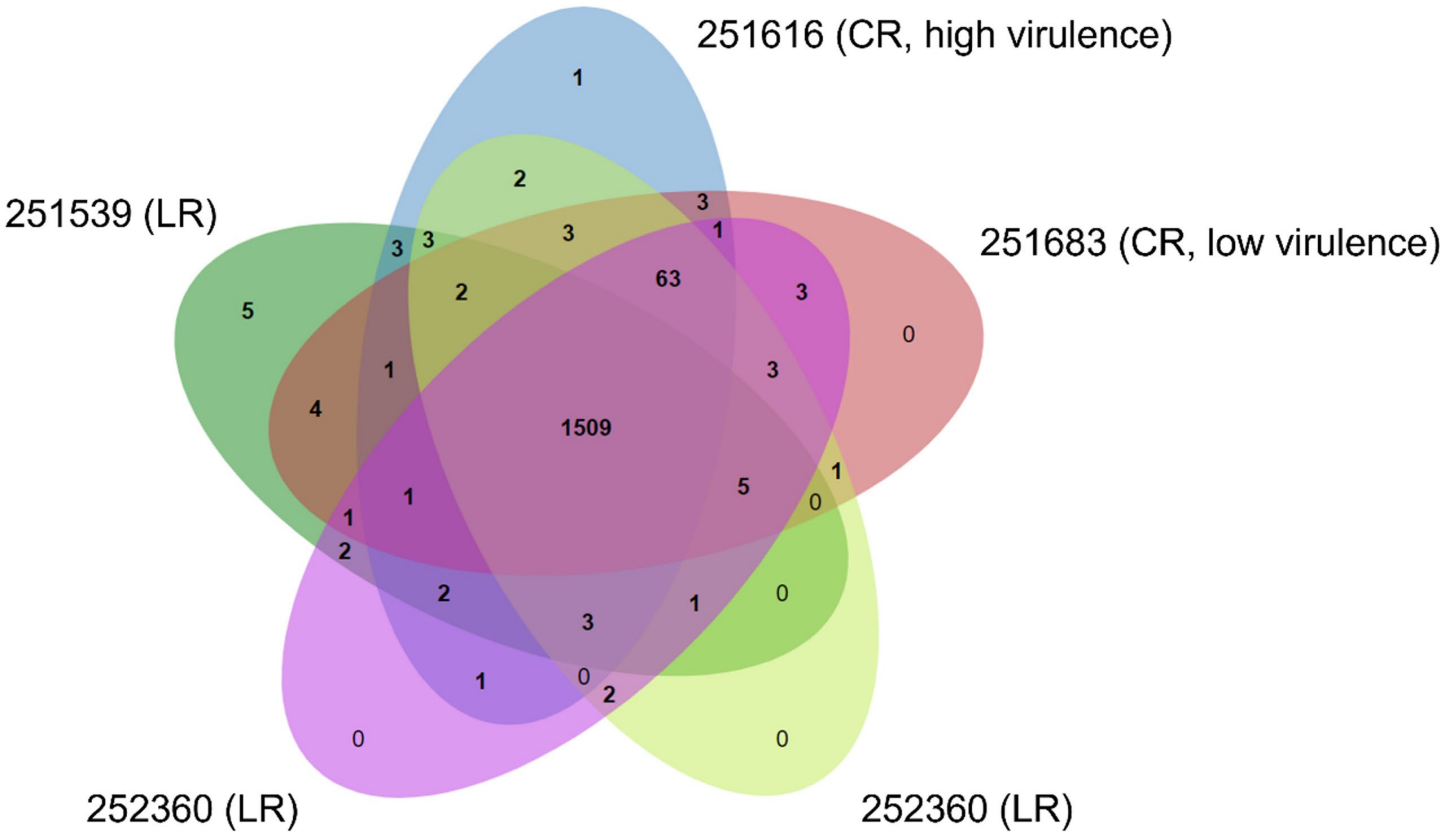
Comparative genomics of the crown- and leather rot pathotypes of *Phytophthora cactorum*. The Venn diagram shows number of shared and unique ortholog clusters of predicted secreted proteins in strawberry crown rot (CR) strains (251616—high virulence; 251683—low virulence) and leather rot (LR) strains (251539, 252360, and 252365).

To identify the secretome associated with the crown- and leather-rot pathotypes of *P. cactorum*, genes encoding putatively secreted proteins in the sequenced genomes were predicted and compared using Orthovenn2 (Table 3; Figure 3).

**Table 3:** The number of predicted cytoplasmic effectors (RxLRs and CRNs), total predicted secretome and apoplastic effectors of the five *Phytophthora cactorum* strains from strawberry.

Strain (pathotype^a^)	Cytoplasmic effectors			Secretome	Apoplastic effectors
	RxLRs^b^	Candidate RxLRs^c^	CRNs^d^		
251539 (LR)	336	222	90	1,797	762
252360 (LR)	303	190	80	1,770	765
252365 (LR)	303	190	83	1,774	766
251683 (CR)	296	188	87	1,786	771
251616 (CR)	309	188	81	1,781	766

a CR, crown rot pathotype; LR, leather rot pathotype.

b RxLR candidates were predicted using the effectR package.

c High confidence RxLR effectors, which means RxLRs with predicted signal peptides.

d Crinkler (CRNs) candidates were predicted using the effectR package.

The predicted secreted proteins from the two CR strains (251616 and 251683) and the three LR strains (251539, 252360, and 252365) of *P. cactorum* formed 1,625 clusters. A total of 1,509 clusters containing 8,238 secreted proteins were common to all *P. cactorum* strains, hereafter referred to as the core *P. cactorum* secretome (Figure 3; Supplementary Table S2). These included genes potentially involved in pathogenesis such as glucan, lignin, pectin, polysaccharide, xylan and xyloglucan catabolic processes (GO:0045490, GO:0000272, GO:0046274, GO:0009251, and GO:2000899, respectively); response to oxidative stress (GO:0006979); pathogenesis (GO:0009405); serine-type endopeptidase and carboxypeptidase activity (GO:0004252); protein with serine/threonine kinase activity (GO:0004674); proteolysis (GO:0006508, GO:0051603); metal ion binding (GO:0046872); cutinase activity (GO:0050525); and cell redox homeostasis (GO:0045454).

To identify genes specific to CR or LR pathotypes, ortholog clusters unique to the LR strains (251539, 252360, and 252365) and the CR strains (251616 and 251683) were analyzed for their potential role in pathogenesis. In the high virulence CR strain 251616, one unique cluster containing two paralogous genes (*251616_g6270* and *251616_g6271*) encoding pectate lyases was detected.

The LR strains and the low virulence CR strain (251683) all contain orthologous genes (*251539_g13496*, *252360_g4306*, *252365_g5402*, and *251683_g8771*) encoding proteins with a fungal-type cellulose-binding domains (CBDs) that formed a cluster. The CBD protein orthologs have sequence similarity to a putative lectin from *P. palmivora* (accession nr. POM66073.1, sequence identity 70%).

The LR strains and the low virulence CR strain (251683) also contain orthologous genes (*251539_g17366, 252360_g7426, 252365_g11583, 251683_g7046*) encoding beta-elicitin proteins that formed a cluster. Using manual BLASTP similar protein sequences were identified in the high virulence CR strain 251616 (*251616_g15069, 251616_g15070*) and also in the previously sequenced CR strains (10300, P414, 15-13, 17-21) and in the apple strains (62471 and R36-14; Armitage et al., 2018; Nellist et al., 2021). Sequence alignment of the beta elicitin protein sequences from the *P. cactorum* strains revealed two single amino acid polymorphisms in the C-terminus, at positions 154 and 170 (Supplementary Figure S1). All the strains contained two alleles of the beta-elicitin gene. In the CR strains 251616 (*251616_g15069, 251616_g15070*), P414 (*Pcac1_g22870, Pcac1_g22871*), and 10300 (*10300_g5489, 10300_g17965*), both alleles (or gene copies) encoded proteins with threonine residues at these positions, T154 and T169. In the LR strains and the low virulence CR strain one or both alleles encoded proteins with methionine and isoleucine at the same positions, M154 and I169 (Supplementary Figure S1; Supplementary Table S3).

In addition, three genes (*251539_g673*, *252360_g9838*, *252365_g11353*) encoding identical protein sequences annotated as small secreted protein with phosphorylation and kinase activity (GO:0016310, GO:0016301) were only detected in the LR strains (Supplementary Table S2).

Comparison of the secretomes of the *P. cactorum* strains showed 63 clusters containing 299 proteins that did not occur in the strain 251539 isolated from apple. Based on the functional annotations, including protein family domain classifications (InterPro) and GO terms, these proteins are involved in several biological processes and molecular functions such as polysaccharide catabolic processes (GO:0000272, GO:0045490, GO:0030245), e.g., 1,4-beta-D-glucan cellobiohydrolase B (IPR001722), pectate and pectin lyases (IPR002022, IPR011050), pectin esterases (IPR000070), polygalacturonases (IPR000743); proteolysis (GO:0006508) including serine-type endopeptidase activity (GO:0004252), e.g., serine proteases with a trypsin domain and chymotrypsin BII (IPR001254) and cysteine-type peptidase activity (GO:0008234), e.g., cysteine peptidase (IPR000668); proteins with serine/threonine kinase activity (GO:0004674), e.g., serine/threonine-protein kinase (IPR008271); pathogenesis (GO:0009405), e.g., avr4-like secreted RxLR effector protein; carbohydrate metabolic process (GO:0005975), e.g., fructose-1,6-bisphosphatase class 1 (IPR044015; IPR033391); lipid catabolism (GO:0016042), e.g., GDSL (Gly-Asp-Ser-Leu) esterase/lipase (IPR036514); fungal-type cell wall organization (GO:0031505), e.g., glycoside hydrolase (IPR004886;); cutinase activity (GO:0050525), e.g., cutin hydrolase (IPR000675) and several proteins with no GO terms (Supplementary Table S2). Of particular interest was a cluster of 20 small cysteine-rich secretory proteins (cluster 9) with 95% sequence similarity to small cysteine-rich secretory protein SCR99 (accession nr. ALC04449.1), which was not detected in the predicted secretome of the apple strain 251539 (Supplementary Table S2).

#### 3.3.3. Genomic variation in *Phytophthora cactorum* strains

**Figure 4:**
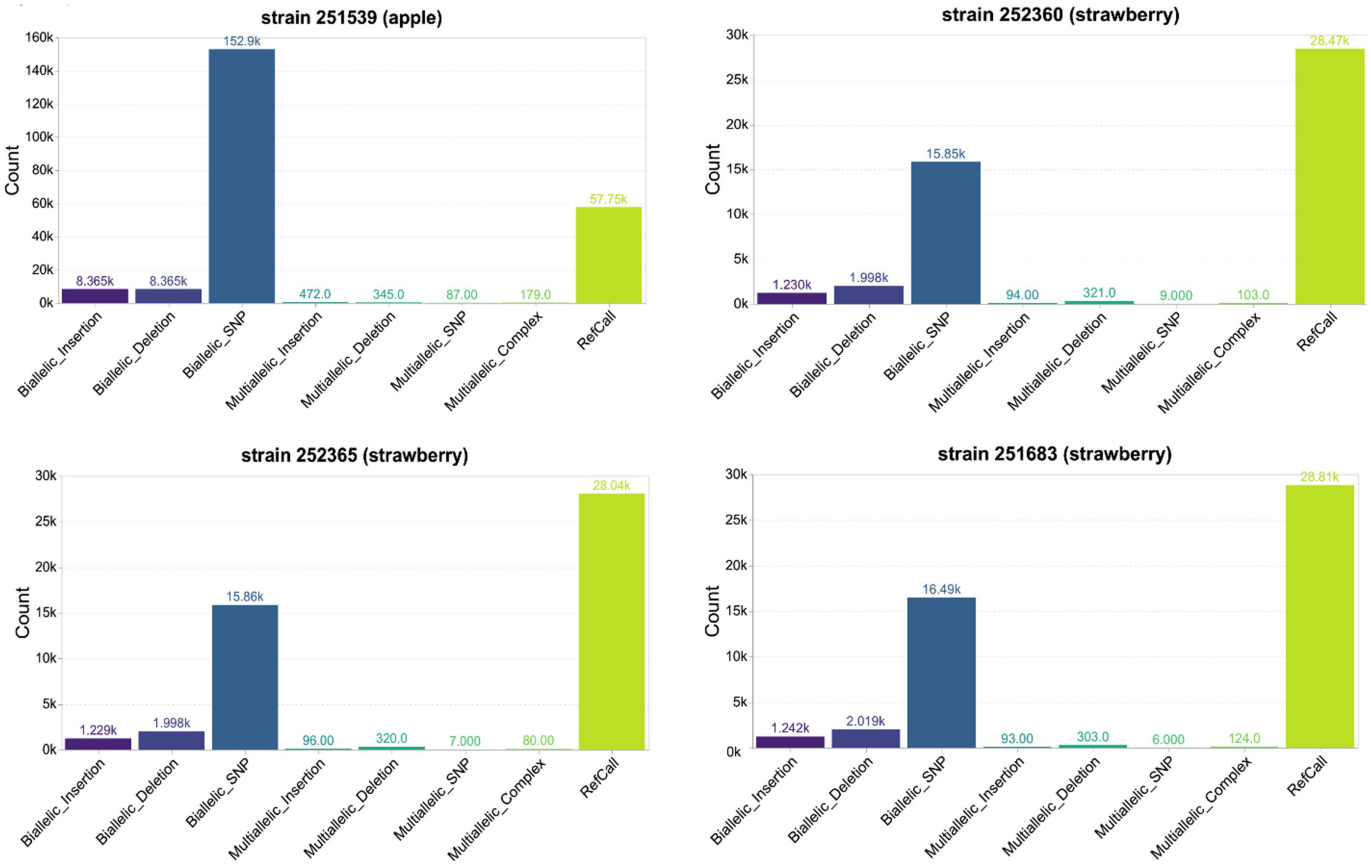
An overview of the variant types detected in the genome of *Phytophthora cactorum* strains from apple and strawberry hosts. Biallelic variants such as single nucleotide polymorphisms (SNPs) and insertions or deletions (Indels) represent genomic variants with one alternate allele detected at a particular site compared to the corresponding site in the reference genome of strain 251616. Multiallelic variants such as multiallelic insertion/deletion/SNP represent more than one alternate allele, while multiallelic complex indicates variants with multiple alternate alleles. Reference call (RefCall) represent candidates that were determined to match the reference. The y-axis shows the count of variants in thousands (*k* = 1,000).

The genome sequence comparisons of the *P. cactorum* LR strains (251539, 252360, 252365) and the low virulence CR strain (251683) with the high virulence CR strain (251616; reference) revealed sequence variations, including single-nucleotide polymorphisms (SNPs) and small insertions or deletions (indels). Most of the SNP and indel calls were biallelic, suggesting that the mycelial stage of *P. cactorum* is diploid. The apple strain 251539 has more genetic variants than the strains isolated from the strawberry host (Figure 4). The total number of high-quality phased SNPs detected in this strain was 5458, which is nearly double the number of SNPs in the LR and low virulence CR strains (Supplementary Table S4). Similarly, the number of high-quality indels in the apple strain 251539 was 15185, which is more than 6-fold the number in the LR and low virulence CR strains (Supplementary Table S5). No phased indels were detected in the *P. cactorum* strains, and thus, haplotype information could not be inferred for the indels.

**Figure 5:**
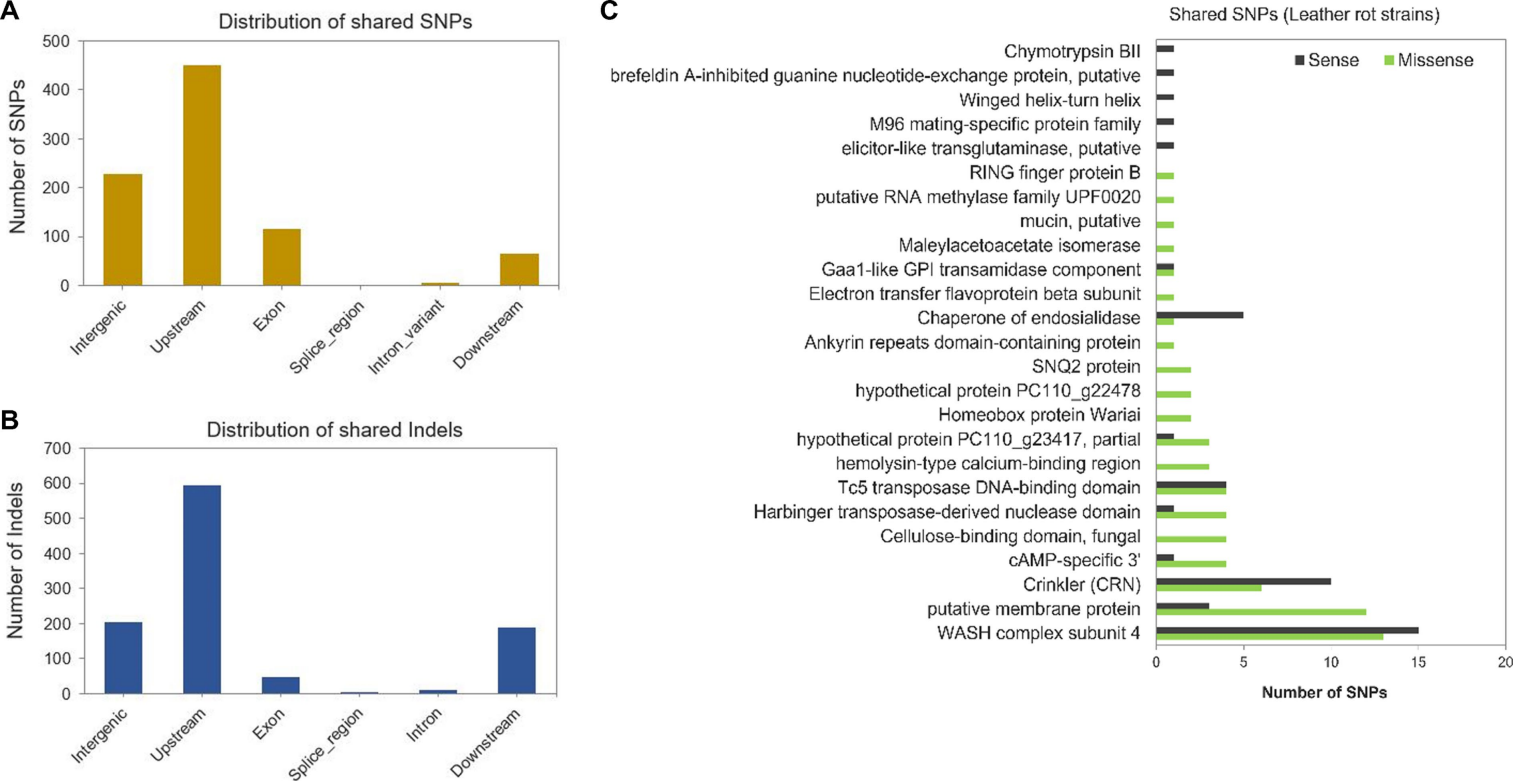
Genomic variants detected in *Phytophthora cactorum* strains. **(A)** Distribution of shared SNPs and **(B)** Indels present in the HiFi reads of leather rot strains 251539, 252360, and 252365 compared to the assembled genome of high virulence crown rot strain 251616 (reference). **(C)** The number of shared synonymous (sense) and nonsynonymous (missense) variants detected in genes belonging to different annotated classes.

To identify genomic variants associated with virulence of *P. cactorum* on strawberry crown, the SNPs and indels detected in the LR strains, 251539, 252360, and 252365, were compared and assigned to the genomic locations in the reference CR strain 251616. Most of the SNPs and indels shared by all LR strains were detected upstream of predicted genes (Figures 5A,B). Forty-six synonymous, 68 nonsynonymous (missense), and two nonsense mutations were detected in the coding sequences (Supplementary Table S4). Nonsynonymous mutations were detected in the genes encoding an ankyrin repeat domain-containing protein (*g10239*), an electron transfer flavoprotein beta subunit (*g344*), a fungal-type cellulose-binding domain-containing protein (*g13858*), a hemolysin-type calcium-binding protein (*g15182*), a homeobox protein Wariai (*g15156*), a hypothetical protein PC110g22478 (*g14092*), a maleylacetoacetate isomerase (*g15372*), a mucin protein (*g87*), an RNA methylase (*g16315*), a RING finger protein (*g16013*), an SNQ2 protein (*g14515*; Figure 5C). More nonsynonymous than synonymous mutations were observed in the genes encoding a membrane protein, a cyclic AMP-specific 3′ protein, a Harbinger transposase-derived nuclease domain protein and a hypothetical protein PC110_g23417 (Figure 5C). To investigate whether these genes were under selective pressure, the rate of nonsynonymous and synonymous mutations per substitution sites (dN/dS or ω) was calculated. The ω was greater than 1 in all the above-tested genes, which signifies positive selection; however, the *p*-values were greater than 0.05 (Supplementary Table S4). Thus, no evolutionary inference could be drawn for the selected genes.

Most of the indels in the coding regions caused frameshift mutations (29%). These were in genes encoding an ankyrin repeat protein, a Crinkler, a Harbinger transposase-derived nuclease domain protein, an RNA methylase, a ribonuclease H-like domain protein, a RING finger protein, a WASH complex subunit 4 protein, a voltage-gated potassium channel subunit beta protein, and in three hypothetical proteins (Supplementary Table S5). In addition, in-frame indels were detected in several genes encoding an annexin family protein, a cellulose-binding domain protein, a Crinkler protein, a cysteine-rich secretory protein, a mucin protein, and a transmembrane protein (Supplementary Table S5).

#### 3.3.4. Predicted RxLR and Crinkler effectors in *Phytophthora cactorum*

**Figure 6:**
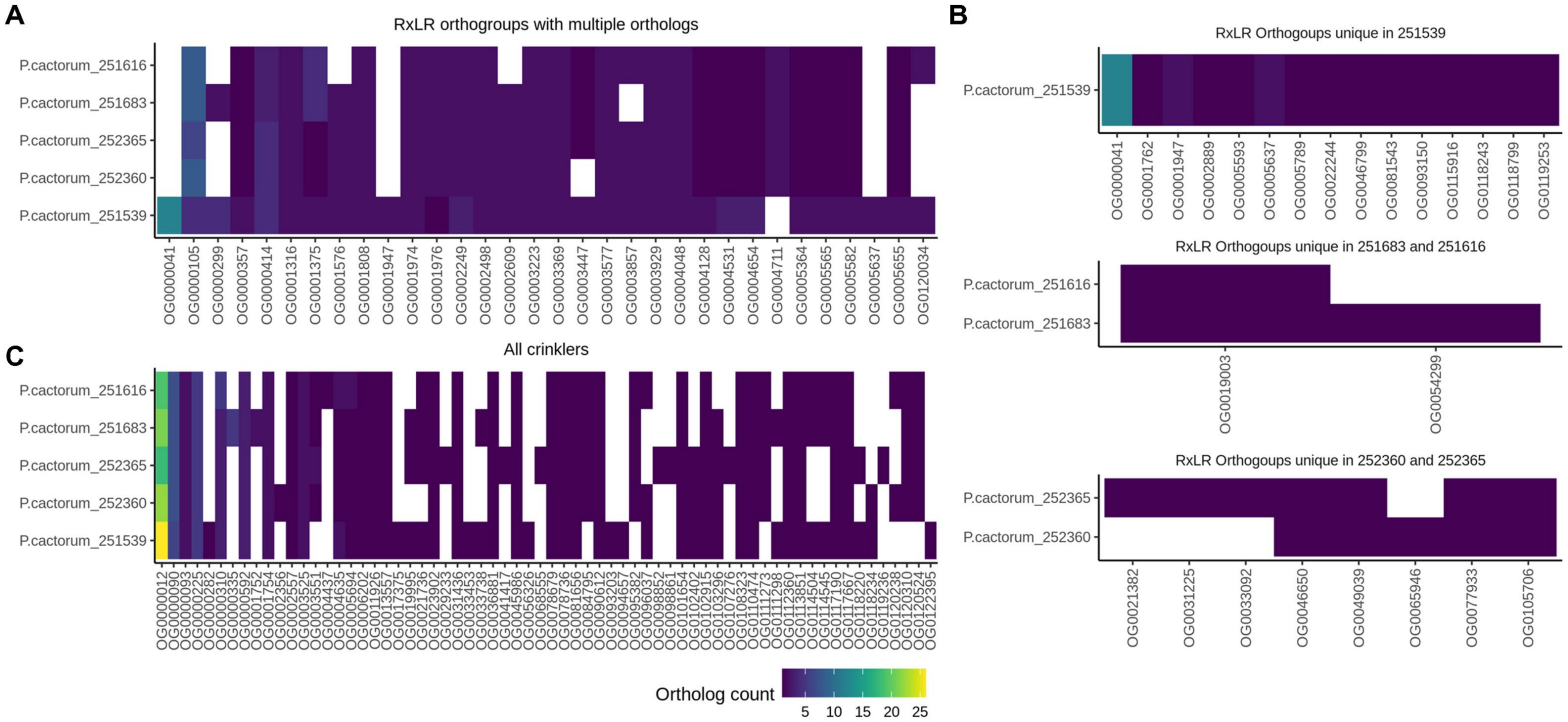
Shared and unique predicted RxLR and Crinkler effector genes in five *Phytophthora cactorum* strains. **(A)** High-confidence candidate RxLRs with multiple gene clusters per orthogroup in the genome of crown rot strains (251616—high virulence and 251683—low virulence) and the leather rot (LR) strains (251539, 252360, and 252365). **(B)** Unique RxLR orthogroups in the apple strain 251539, unique orthogroups in the crown rot and leather rot strains. **(C)** Orthogroups containing predicted Crinkler effectors in the genome of *P. cactorum* strains. Each column represents an orthogroup. The color gradient represents number of RxLR and Crinkler genes per genome.

The initial prediction of candidate RxLR effectors with the RxLR-ERR motif and Crinklers (CRNs) with the LFLAK-HVLV motif using regular expressions (regex) and a Hidden Markov Model (HMM) resulted in varied numbers of candidate effectors in the five sequenced *P. cactorum* strains (Table 3). Most of the predicted CRN effectors lacked a conventional signal peptide in the N-terminus. The prediction tool Phobius predicted more CRNs with a signal peptide than SignalP5 (Supplementary Table S6). Thus, all CRN effectors with or without a signal peptide were considered for further analysis. To identify candidate effectors involved in the infection of the strawberry rhizome, predicted high-confidence RxLR and CRN effectors from the CR and LR strains were compared. The comparison of RxLR protein sequences revealed several orthogroups that contained multiple copies of RxLRs, and notably differences in copy number between the CR and LR strains (Figure 6; Supplementary Figure S2). A higher number of RxLRs in orthogroup OG0000414 was observed in the LR strains than in the CR strains. This orthogroup, contained a gene encoding a protein with similarity to the *P. palmivora* avirulence-like protein Avr1b-1 (accession nr. POM73746.1, 74.5% sequence identity). By contrast, the orthogroup OG0001375 contained more RxLR copies in the CR strains than the LR strains.

No RxLR candidates were identified that were unique to the CR strains. Conversely, the LR strains contained an orthogroup OG0024976 with a single gene, which was not found in the CR strains. In addition, all LR strains and the low virulence CR strain (251683) contained orthogroups OG0001576, OG0002609, OG0005599, and OG0057065, which was not detected in the high virulence CR strain 251616 (Figure 6A; Supplementary Table S6). Several unique orthogroups of RxLRs were also identified in the apple strain 251539 (Figure 6B).

The comparison of CRN effectors of *P. cactorum* strains showed a similar pattern of expansion and contraction of genes in certain orthogroups as the RxLRs (Figure 6C). CR strains contained a higher number of CRN genes in orthogroups OG0000310 and OG0005694 than in the LR strains. Furthermore, the high virulence crown rot strain contained an orthogroup OG0004437 with a single CRN gene *251616_g2371* that was unique to this strain (Figure 6C; Supplementary Table S6).

## 4. Discussion

### 4.1. Highly contiguous and accurate genome assemblies of *Phytophthora cactorum*

Highly contiguous genome assemblies provide a better framework to understand the biology of the studied organisms by offering better information on the genomic context of traits of interest. In this work, long HiFi reads facilitated high-quality genome assemblies of the five sequenced *P. cactorum* strains. The total number of contigs ranged from 178 to 203, which was a major improvement from the 4,623 contigs of our previously sequenced *P. cactorum* 10300 genome assembly and 3644-20136 contigs of other publicly available *P. cactorum* genome assemblies (Grenville-Briggs et al., 2017; Armitage et al., 2018; Nellist et al., 2021). The genome sizes of the *P. cactorum* strains sequenced here were similar to the 10300 assembly and several other *P. cactorum* assemblies recently reported (Nellist et al., 2021), and therefore, according to expectations. The high number of contigs and low N50 value in the Yunnan assembly might help to explain the unexpectedly large genome size of 121.5 Mb and a distinct clade in the phylogenomic analysis in this work (Figure 2A). The previously sequenced genome of strain P414 (deposited as a reference genome in NCBI) assembled into 194 contigs (Nellist et al., 2021), which is in the range of our five newly sequenced genomes reported here. However, the new genome assemblies have 38–60% higher (and better) N50 value than the P414 assembly, which likely improves genome quality, particularly in regions of repetitive sequences (Table 2).

### 4.2. Genomic variations between crown rot and leather rot pathotypes of *Phytophthora cactorum*

The whole genome phylogenomic study showed that *P. cactorum* strains from the strawberry rhizome or fruit tissues were genetically more similar than the apple strain 251539. The result was consistent with the variant analysis that identified a higher number of SNPs and small indels in the apple strain than the *P. cactorum* strains from strawberry host. A similar study showed that *P. cactorum* from strawberry and apple hosts were genetically two distinct population that formed two non-recombining clades for strawberry CR and apple strains (Nellist et al., 2021). The present study supports the previous findings that describe *P. cactorum* as species complex and should not be considered as generalist pathogen.

Understanding the mechanisms of plant recognition of pathogen molecules (PAMPs/MAMPs) and identifying virulence determinants of the pathogen is essential to develop effective strategies to control strawberry diseases. The comparative genomic analyses of the crown- and leather-rot strains revealed genes encoding proteins potentially involved in pathogenesis, with sequence variations and presence or absence polymorphisms. The presence of two unique pectate lyase genes (*251616_g6270* and *251616_g6271*) in the CR strain 251616 might contribute to the virulence in the strawberry crown. Interestingly, the homologous gene *Pcac1_g14072* of the previously sequenced CR strain P414 was shown to be upregulated during early infection in strawberry (Nellist et al., 2021). Pectate lyases secreted by plant pathogens are known to degrade networks of pectin present in the plant cell wall. They cleave the glycosidic bonds of two saccharide units via a β-elimination mechanism (Collmer and Keen, 1986; Herron et al., 2000). Studies have shown that pectate lyases contribute to virulence of bacteria, fungi, nematodes, and oomycetes (Wegener, 2002; Fu et al., 2013; Cho et al., 2015; Chen et al., 2021). A study of pectate lyase genes *PL1*, *PL15*, *PL16*, and *PL20* from *P. capsici* showed that overexpression of these genes in a mildly virulent strain transformed it to a highly aggressive strain (Fu et al., 2015). Another study demonstrated that deletion of a pectate lyase gene *PEL1* in the fungal pathogen *Verticillium dahliae* compromised virulence in *Nicotiana benthamiana* and cotton plants (Yang et al., 2018b). The two pectate lyases genes in the high virulence CR strain (*251616_g6270* and *251616_g6271*) are promising candidates to further study *P. cactorum* virulence in strawberry.

A unique cluster of putative CBD proteins was observed in all the LR strains. Previous studies suggested that CBD proteins are involved in oomycetes cell wall development and interacts with plant cell wall components (Gaulin et al., 2006; Jones and Ospina-Giraldo, 2011). The presence of CBD proteins at the cellular surface of *Phytophthora* facilitates binding to cellulosic substrates (Gaulin et al., 2002). In plants, alteration of cellulose in the plant cell wall is a warning signal to activate defense responses (Dumas et al., 2008). Although, an increased accumulation of cytosolic calcium was reported in response to CBEL in tobacco cells (Dumas et al., 2008), the exact mechanism of plant defense is unknown. Studies have shown that *Phytophthora* CBD proteins with lectin-like activities such as CBELs act as potent elicitors of plant defense (Mateos et al., 1997; Gaulin et al., 2006). In this study, the CBD protein-coding genes (*251539_g13496*, *252360_g4306*, *252365_g5402*, and *251683_g8771*) was of particular interest as these were identified in a cluster unique to the LR strains and the low virulence CR strain. A similar CBD gene (*251616_g13858*) with four nonsynonymous but no synonymous SNPs was detected in the high virulence CR strain, reflecting signs of positive selection or result of functionally neutral mutation fixed due to drift.

In addition to the variations in the CBD proteins between the high virulence CR strain and the LR strains (including the low virulence CR strain), two single amino acid polymorphisms (T154M and T169I) were observed in the C-terminus of a beta elicitin protein. Interestingly, threonine residues were conserved in both the alleles of the beta elicitin in the high virulence CR strain 251616 and in the previously sequenced CR strains 10300 and P414, except in the strains 15–13 (M^154^, I^169^) and 17–21 (M^154^, T^169^; Supplementary Table S3). All LR strains from strawberry fruit and apple including the low virulence CR strain 251683 contained one or both alleles with methionine and isoleucine at these positions. Many *Phytophthora* elicitins are rich in threonine, serine and proline residues at their C-terminal domain and these residues are suggested to be involved in association with the cell wall through extensive glycosylation (Jiang et al., 2006). Elicitins are often recognized as PAMPs by the host defense machinery, possibly by immune receptors such as BRI1-associated receptor kinase 1 (BAK1) and cell wall-associated receptor-like kinase 1 (WAK1) (Chaparro-Garcia et al., 2011; Raaymakers and Van den Ackerveken, 2016). Pernollet (1993) studied the role of threonine residues in α-elicitin from *P. cactorum* and proposed that the replacement of a lysine residue by threonine is partly responsible for reduced necrotic activity of the α-elicitin, possibly by avoiding the host defense machinery (Pernollet, 1993). Similar peculiarities were observed in a beta elicitin protein sequence from *P. megasperma* that was less toxic than related proteins from other *Phytophthora* spp. (Huet and Pernollet, 1993). Furthermore, cysteine-rich secretory proteins encoded by the SCR-108-like gene cluster detected in the LR strains are interesting candidates for host recognition as similar SCR (e.g., SCR96) of *P. cactorum* was reported to induce cell death responses in other host plants (Chen et al., 2016). Further study is needed to examine if the putative elicitor genes such as *CBD*, *beta elicitin* and *SCR-108-like* from the LR strains are expressed during interaction with strawberry. It is worth mentioning that the expression of the beta elicitin genes (*Pcac1_g22870, Pcac1_g22871*) from the CR strain P414 were upregulated during strawberry infection in the transcriptome dataset previously reported by Nellist et al. (2021).

Several genes encoding annexin family members, Crinklers, cysteine-rich secretory protein, a mucin protein, a putative transmembrane protein had sequence variation in the LR strains compared to the high virulence CR strain. Some of the gene products from these, such as an annexin family protein and mucin have been reported to be associated with the cell surface in the close relative *P. infestans* (Grenville-Briggs et al., 2010). Larousse et al. (2014) studied the role of mucin-like genes in *P. parasitica* and found that their gene products accumulated on the surface of biofilms, which favor attachment and promote virulence through aggregation. Thus, sequence variations in these genes might affect *P. cactorum* tissue specific interaction or pathotype.

The high virulence CR strain may possess altered recognition domains or have lost potential elicitors as a mechanism to escape host defense. Since the LR strains were able to cause disease in strawberry fruits after inoculation, but not in the rhizome of the same cultivar, led us to question whether activation of host defense machinery was tissue specific. Eikemo and Stensvand (2015) studied the resistance level of both the tissues (rhizome and fruit) of the same strawberry cultivars against crown rot and leather rot. They reported that cultivars most robust to leather rot was susceptible to crown rot and vice versa. Thus, the mechanisms for resistance against the two diseases seem to be different on the two tissues types. Casado-Díaz et al. (2006) studied the spatial expression pattern of defense-related genes in strawberry during infection with *Colletotrichum acutatum*, a hemibiotrophic fungal pathogen. They reported that expression of a leucine rich repeat receptor-like protein-coding gene *Falrrk-1* and a chitinase gene *Fachit-1* was strongly downregulated in infected fruit tissue, while their expression increased several folds higher in the infected rhizome (crown) tissue. They also found that strawberry fruit was more susceptible to *C. acutatum* infection than crown tissues. Thus, host-pathogen interactions may be influence by tissue architecture and/or differential expression of defense-related genes. The identification of potential elicitor genes of *P. cactorum* warrants further investigation to understand the virulence mechanisms of the CR and LR pathotypes in strawberry. In addition, several secreted protein-coding genes were detected only in the genome of *P. cactorum* strains from strawberry host and not present in the genome of apple strain. Most of these genes encode proteins that have a putative role in pathogenesis, suggesting co-evolution and acquisition of pathogenesis-related genes during host adaptation as observed in other pathosystems (Gaulin et al., 2018; Jangir et al., 2021).

The new genome assemblies of *P. cactorum* identified a greater number of predicted RxLRs via the REGEX and HMM models than previously reported for other strains of the species (Armitage et al., 2018; Yang et al., 2018a; Nellist et al., 2021). Remarkably, most of the predicted CRNs lacked a conventional signal peptide in the N-terminus. It has been proposed that CRN effectors that lack a conventional signal peptide might translocate into host cells via an unconventional secretion pathway (Meijer et al., 2014; Amaro et al., 2017; Voß et al., 2018). The number of predicted CRN effectors was in agreement with the previously published genomes from *P. cactorum* strains (Armitage et al., 2018; Nellist et al., 2021) and *P. palmivora* P16830 (Evangelisti et al., 2017).

The analysis of the predicted RxLR and CRN effectors provided evidence for expansion and contraction of certain gene clusters in the investigated *P. cactorum* strains (Figure 6). In the high virulence CR strain, the unique CRN gene *251616_g2371* and the expansion of CRNs genes in the orthogroups OG0000310 and OG0005694 need further investigation for their virulence function in the *P. cactorum*-strawberry interaction. In addition, the loss of RxLR genes in orthogroup OG0000414 in this strain should be considered for future functional studies, if they are expressed during infection of strawberry. Among the reduced effector genes in this strain was an RxLR gene with sequence similarity to *Avr1b-1* from *P. palmivora*. Gene loss has been recognized as an important mechanism for emergence of virulence in bacterial, fungal, oomycete pathogens (Maurelli, 2007; Qutob et al., 2009; Bliven and Maurelli, 2012; Rouxel and Balesdent, 2017; Siscar-Lewin et al., 2019). For instance, loss of *Avr-Co39* in rice-infecting haplotypes of *Magnaporthe oryzae* has been proposed as an adaptive mechanism to escape recognition by the R protein Pi-Co39 (Couch et al., 2005). However, loss or mutated effectors may impose a fitness penalty on the pathogen (Leach et al., 2001; Montarry et al., 2010). The functional redundancy of paralogous genes of RxLRs of *P. cactorum* could play a potential role in conserving virulence function and fitness, as addressed for bacterial and oomycete effectors during pathogenesis (Birch et al., 2008; Ghosh and O’Connor, 2017). Nellist et al. reported that over half of the candidate effectors were not expressed or showed low expression *in planta* (Nellist et al., 2021). Thus, further expression analysis is required to pinpoint candidate effectors involved in tissue-specific interactions of *P. cactorum* in strawberry.

## 5. Conclusion

The comparative genome analysis identified sequence variations between the studied CR and LR strains, which might contribute to the tissue-specific interactions of *P. cactorum* in strawberry. The presence of two unique pectate lyase genes, and expansion of potential virulence effector genes such as *RxLRs* and *CRNs* in orthogroups OG0001375, OG0000310, and OG0005694 or loss of avirulence determinants (e.g., *Avr1b-1-like* gene) in the orthogroup OG0000414 may play a role in increased virulence of the CR strain 251616 in the strawberry rhizome. However, functional analysis is required to validate these genes as pathogenicity determinants of *P. cactorum* in strawberry tissues. The non-virulence of the LR strains in the rhizome tissues might be an event of recognition of elicitors by host proteins. The data presented here can be used as a basis for future functional analysis of potential effector genes of *P. cactorum* during interaction with strawberry.

## Data availability statement

The data presented in the study are deposited in the DDBJ/ENA/GenBank repository under the BioProject PRJNA885305, and under the accessions JAPDOL000000000, JAPDOM000000000, JAPDON000000000, JAPDOO000000000, JAPDOP000000000. The raw sequence reads are deposited in the NCBI Sequence Read Archive (SRA) under the accessions SRR21764265, SRR21764266, SRR21764267, SRR21764268, SRR21764269.

## Author contributions

AG and AS performed the inoculation experiment and data analysis. AG, SR, and EL analyzed the sequencing data. MB and AS formulated the research idea and designed the experimental setup. AG interpreted the results and wrote the first draft of the manuscript. All authors contributed to the article and approved the submitted version.

## Funding

AG was funded by a PhD scholarship from the Norwegian University of Life Sciences, Ås, Norway. The work was supported by NIBIO (basic funding) and the Research Council of Norway, grant number 326212.

## Conflict of interest

The authors declare that the research was conducted in the absence of any commercial or financial relationships that could be construed as a potential conflict of interest.

## Publisher’s note

All claims expressed in this article are solely those of the authors and do not necessarily represent those of their affiliated organizations, or those of the publisher, the editors and the reviewers. Any product that may be evaluated in this article, or claim that may be made by its manufacturer, is not guaranteed or endorsed by the publisher.

## Footnotes

^1^https://github.com/PacificBiosciences/pbmm2

^2^https://github.com/lastz/lastz
