# Supplementary material for: Genome analysis of Phytophthora cactorum strains associated with crown- and leather-rot in strawberry
Source: Front Microbiol. 2023 Jul 3;14:1214924. doi: 10.3389/fmicb.2023.1214924 (PMC10351607; doi:10.3389/fmicb.2023.1214924)
Supplement: Supplementary file 1 [file Data_Sheet_1.docx]

Genome analysis of *Phytophthora cactorum* strains associated with crown- and leather-rot in strawberry

Supplementary Material

Supplementary Figures


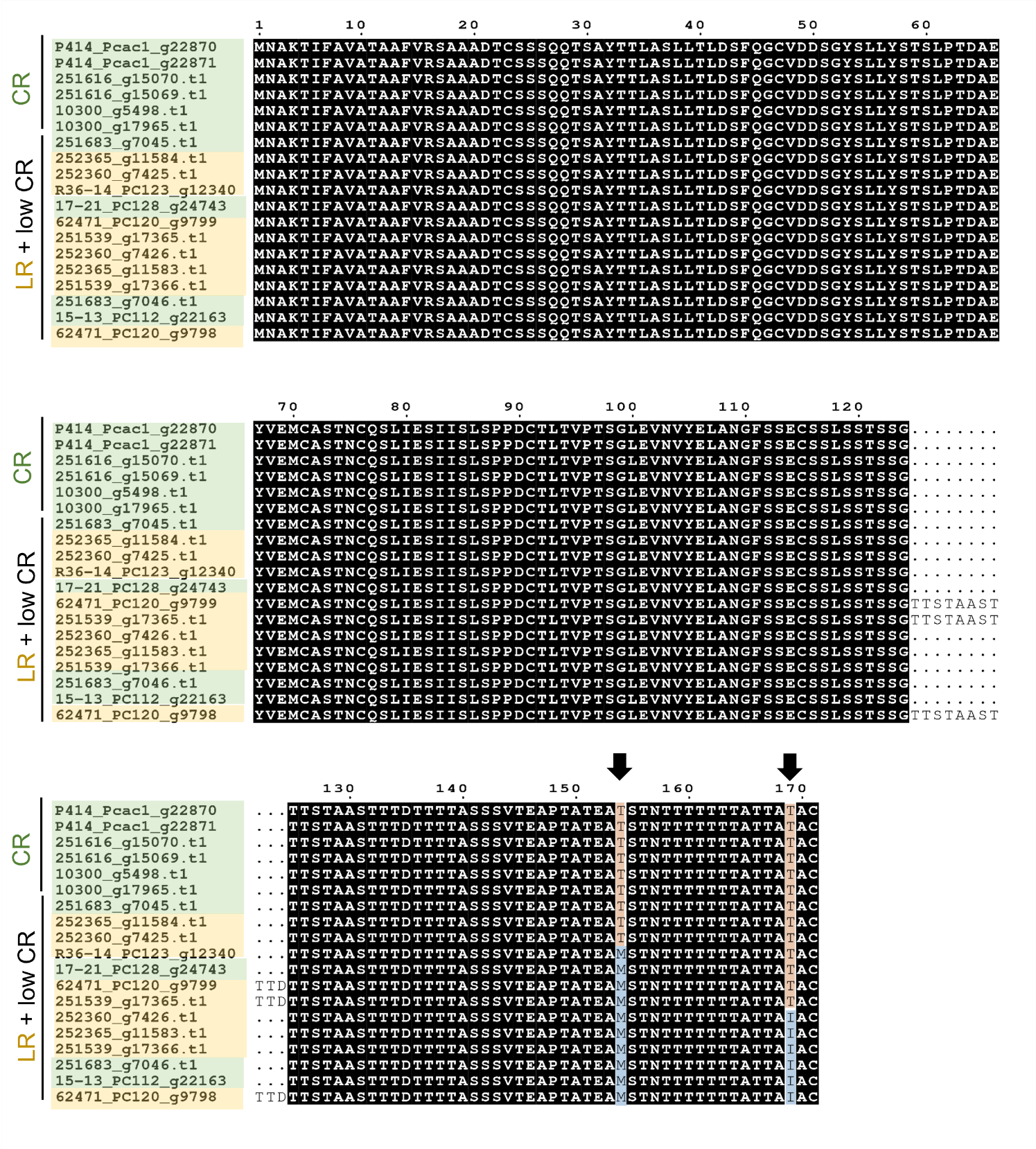


**Supplementary Figure 1.** Multiple sequence alignment of the beta-elicitin DRE proteins from different *Phytophthora cactorum* strains, two variants from each strain. Proteins from the crown rot (CR) strains (251616, P414, 10300, and 15-13) including the low virulence strains (251683 and 17-21) are highlighted in light green, whereas the leather rot (LR) strains (251539, 252360, 252365, R36-14, and 62471) are highlighted in light orange. Black arrows on top of the alignment represent amino acid polymorphisms.


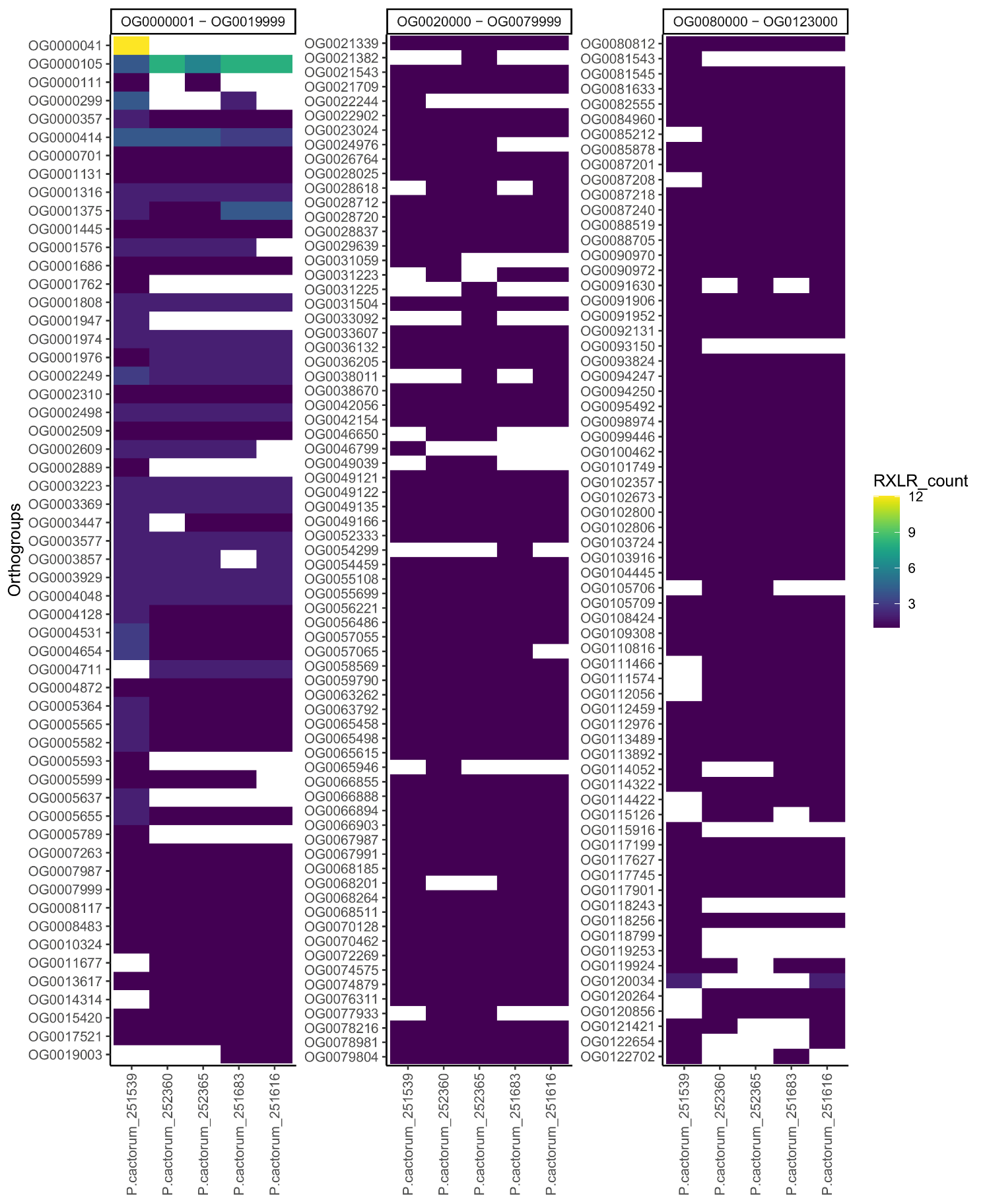


**Supplementary Figure 2.** Clustering of the total predicted high-confidence candidate RxLR effectors of *Phytophthora cactorum* strains. Each column represents an orthogroup. The color gradient represents number of RxLR genes per genome.
