# Supplementary material for: Genome analysis of Phytophthora cactorum strains associated with crown- and leather-rot in strawberry
Source: Front Microbiol. 2023 Jul 3;14:1214924. doi: 10.3389/fmicb.2023.1214924 (PMC10351607; doi:10.3389/fmicb.2023.1214924)
Supplement: Supplementary file 4 [file Table_3.DOCX]

**Supplementary Table 3.** Amino acid polymorphisms of the beta elicitin protein from the crown rot (CR) and leather rot (LR) strains of *Phytophthora cactorum*.

| Strain | Pathotype | Material isolated from | Polymorphism in the beta elicitin protein at amino positions 154 and 169 | | Previously reported |
| --- | --- | --- | --- | --- | --- |
|  |  |  | Allele 1 | Allele 2 |  |
| 251616 | CR | Strawberry (rhizome) | T^154^, T^169^ | T^154^, T^169^ |  |
| P414 | CR | Strawberry (rhizome) | T^154^, T^169^ | T^154^, T^169^ | Nellist et al. 2021 |
| 10300 | CR | Strawberry (rhizome) | T^154^, T^169^ | T^154^, T^169^ | Armitage et al., 2018 |
| 15-13 | CR | Strawberry (rhizome) | M^154^, I^169^ | - | Nellist et al. 2021 |
| 215683 | CR (low) | Strawberry (rhizome) | M^154^, I^169^ | T^154^, T^169^ |  |
| 252360 | LR | Strawberry (fruit) | M^154^, I^169^ | T^154^, T^169^ |  |
| 252365 | LR | Strawberry (fruit) | M^154^, I^169^ | T^154^, T^169^ |  |
| 17-21 | CR (low) | Strawberry (fruit) | M^154^, T^169^ | - | Nellist et al. 2021 |
| 251539 | LR | Apple (fruit) | M^154^, I^169^ | M^154^, T^169^ |  |
| 62471 | LR | Apple | M^154^, I^169^ | M^154^, T^169^ | Nellist et al. 2021 |
| R36-14 | LR | Apple | M^154^, T^169^ | - | Nellist et al. 2021 |
